# Causal assessment of smoking and tooth loss: A systematic review of observational studies

**DOI:** 10.1186/1471-2458-11-221

**Published:** 2011-04-08

**Authors:** Takashi Hanioka, Miki Ojima, Keiko Tanaka, Keitaro Matsuo, Fumihito Sato, Hideo Tanaka

**Affiliations:** 1 Department of Preventive and Public Health Dentistry, Fukuoka Dental College, Fukuoka, Japan; 2 Department of Preventive Dentistry, Graduate School of Dentistry, Osaka University, Osaka, Japan; 3 Department of Public Health, Faculty of Medicine, Fukuoka University, Fukuoka, Japan; 4 Division of Epidemiology and Prevention, Aichi Cancer Center Research Institute, Nagoya, Japan

## Abstract

**Background:**

Tooth loss impairs oral function. The aim of the present review was to evaluate the causal association between smoking and tooth loss on the basis of high-quality studies.

**Methods:**

Relevant literature was searched and screened, and the methodological quality was assessed. Information on the strength of the association between smoking and tooth loss, the dose-response relationship and natural experimental data was collected and evaluated with respect to consistency and study design.

**Results:**

Our literature search yielded 496 citations, and 6 cross-sectional and 2 cohort high-quality studies examining 58,755 subjects in four countries. All studies reported significant associations, although the strength of the association was usually moderate. Four studies reported dose-response relationships between exposure to smoking and the risk of developing tooth loss. A decrease in the risk of tooth loss for former smokers was evident in six studies. Interpretation of evidence for each element was consistent, despite some shortcomings regarding study type and population.

**Conclusions:**

Based on the consistent evidence found with the existing biological plausibility, a causal association between smoking and tooth loss is highly likely. Further studies using a cohort design and different populations are necessary to confirm this association.

## Background

Evidence supporting a causal association between smoking and periodontal disease has been accumulated by epidemiological and basic studies over the past two decades. Periodontal disease is now considered a disease group and there is sufficient evidence to infer its causal association with smoking [1].

Among the negative effects of smoking on health, special attention should be given to the treatment outcomes of oral diseases. A negative response to periodontal treatment is consistently reported [2,3]. Furthermore, more frequent recurrence of periodontal disease in smokers than in non-smokers during periodontal maintenance has also been reported [4,5], and an association between smoking and tooth loss during this period has recently been reported [6]. Evidence regarding the effects of smoking on periodontal disease and treatment indicates that smokers lose more tooth-supporting tissue than non-smokers.

Numerous studies have been conducted regarding the association between smoking and tooth loss. Because randomised controlled design studies on smoking are unethical, previous studies on smoking have been observational. Tooth loss may not appear in the latent period of exposure in the way of occurrence of smoking-related death [7]. Smoking is generally prevalent in developed countries, and thus the study population may have been restricted. Tooth loss as a study outcome is an irreversible event; in other words, it is cumulative in extent and prevalence. The definition of tooth loss may vary according to the age of the study population. Therefore, results from these studies should be carefully analysed and interpreted with respect to methodological heterogeneity.

To our knowledge, a causal association between smoking and tooth loss has not been evaluated because of the need for studies that adopt a rigorous approach to validating causality and problems in assessing the quality of studies when extracting the evidence. This review focuses on validating the causal association between smoking and tooth loss according to guidelines for reporting evidence of observational studies [8] and by evaluating the methodological quality of the studies [9]. The primary question of the present review is, 'Does smoking cause tooth loss?'

## Methods

### Literature search

An electronic search was conducted to identify relevant literature. The databases used for the literature search were Medical Literature Analysis and Retrieval System Online (MEDLINE), EMBASE and the Cochrane Central Register of Controlled Trials (CENTRAL). These were searched for papers and abstracts published up to May 2010. Our search strategy was originally developed for application to MEDLINE (via PubMed), and the terms used were [(smoking) OR (tobacco) AND (tooth loss)]. We used [smoking AND (tooth loss OR tooth extraction)] for additional searches of EMBASE (limited to title) and CENTRAL (limited to title, abstract or keywords).

After initial screening, we further conducted a hand search to avoid the omission of recently published studies (June 2008-May 2010). From the results of the initial screening, we identified eight journals that published relevant articles (see Additional file 1). Any potential studies in the reference lists of the identified articles read completely were also considered.

### Categories of outcome and exposure

The primary outcome of interest was tooth loss. For individual oral health, periodontal disease and dental caries may be added to tooth loss as an outcome measure of exposure to smoking. However, tooth loss was the only variable accepted as an outcome variable in the present review. We used three categories for the exposure criterion: current smoker, former smoker and non-smokers.

### Eligibility criteria and screening process

The inclusion criteria for studies were as follows: published in English, investigated associations between smoking and tooth loss and reported the effect size of the association (i.e. literature that employed the variable of smoking only for adjustment and which did not report the effect size of smoking was excluded).

We excluded literature reviews from the search. Furthermore, studies that defined tooth loss using measures other than the two categories were excluded because the definition of tooth loss did not comply with the standard for evaluation of strength of association. We further excluded studies that combined former smokers with non-smokers or current smokers because the causal association between smoking and tooth loss may have been diluted.

Search results were stored using literature management software (iPubMedMaker 7, Sapporo, Japan) for initial screening on the basis of the title and abstract. Two calibrated reviewers screened the results independently. Disagreements between reviewers were resolved by discussion until a consensus was reached. Final screening consisted of the evaluation of full-text reports.

### Methodological quality assessment

Two reviewers independently assessed the methodological quality of studies using the modified Newcastle-Ottawa Scale (NOS) for observational studies. NOS is comprehensive and has been partly validated [9]. The original NOS assessed each criterion for eight items regarding the methodology of observational studies, and a study was awarded 'yes' for each criterion that was clearly satisfied. The grouping items of NOS comprised three categories: selection, comparability and exposure/outcome measurement.

Because the original NOS is comprehensive, we modified the scale for this review. For cross-sectional studies, one star was given to a study for each of the following items in the selection category that were satisfied: validation of the number of teeth by health professionals, definition of tooth loss with the number of lost teeth and the representativeness of the sample of the population. A maximum of two stars was given in comparable categories assessing any possible confounder: one star was given if a study was adjusted for age and one more was given if at least one variable each for socioeconomic status and oral health behaviour was satisfied. One star was given to the item ascertaining exposure and employment of a secure record or structured interview where the researcher was blind to case/control status.

The methodological quality of the cohort studies was also evaluated in three categories. One star was given to each item in the category of selection: representativeness of the sample in the community and the use of a secure record or structured interview where the researcher was blind to the case/control status for ascertaining exposure. Items in comparable categories were evaluated with the same criteria used for the cross-sectional study. One star was given to each item in the category of outcome: validation of the number of teeth by health professionals, follow-up period of one year or more and validation of the similarity of the dropout rate in exposure and control groups of less than 20% of the rate.

Cross-sectional and cohort studies were given six and seven stars, respectively, if the study satisfied all items. Two reviewers independently coded the items in the modified NOS. Disagreements between reviewers were resolved by discussion until a consensus was reached. Indistinct issues were resolved by consultation with a third reviewer. The codes of studies authored by the reviewers were verified by a different reviewer. According to the total number of stars, overall quality was evaluated as follows: five or more stars for high-quality studies, three or four stars for moderate-quality studies and two or fewer stars for low-quality studies.

### Data abstraction

Data on the following elements were abstracted from the studies searched by one reviewer and verified independently by another reviewer. Disagreements between reviewers were resolved by discussion until a consensus was reached. The abstracted elements are shown in Table 1.

**Table 1:** Elements abstracted from searched studies

Study	Elements
All studies	Citation and publication status
	Study design: cross sectional or cohort study
	Participants: number, sex, age range, country, residency and representativeness
	Focal factor(s) with respect to the association with tooth loss: smoking only or various factors including smoking
	Factors entered in the final analytical model
	Type of the estimate of association, effect size and confidence interval
	Category of evaluated group: age group, sex and type of exposure
	Statistical significance of the dose-response relationship
	Special mention: sensitivity, subgroup and other types of analyses and the source of funding
Cross sectional studies	Definition and prevalence of tooth loss
Cohort studies	Observational length and non-respondent and follow-up rates

### Analysis for causality

Several methods have been proposed to evaluate the causal association of factors for a multi-factorial disease. In this review, three elements were extracted and used according to the Bradford Hill criteria [10] and a Surgeon General Report [1] as follows: the strength of association (magnitude and its statistical strength), biological gradient (dose-response relationship) and natural experiment. Consistency of abstracted data was also assessed to allow the synthesis of evidence for each element. Biological plausibility (coherence and analogy) was considered with respect to the effects of smoking on periodontal tissue breakdown, in addition to the epidemiological pattern of tooth loss and the tobacco epidemic. We did not address temporality, which refers to the generally apparent sequence of smoking and tooth loss.

Common descriptors for the strength of association were defined using the effect size as follows: ≤1.49 for a weak association; 1.50-2.99 for a moderate association; and ≥3.00 for a strong association [11]. Qualitative evaluation of the strength of association was performed using differences in percentages (points) between case and control groups when the prevalence of tooth loss exceeded 15% as follows: ≤6.9 points for a weak association, 7.0-19.9 points for a moderate association and ≥20.0-points for a strong association.

The element of dose-response relationships was summarised according to descriptions in each study, and if available, the statistical significance of the correlation. The Bradford Hill criterion of the experiment was evaluated by comparing the strength of association between former and current smokers relative to non-smokers. This criterion was named 'natural experiment' [1], because interventional studies are difficult to conduct in humans. The statistical significance of the strength of association in former smokers was also evaluated, although the control group in this comparison was consistently set for non-smokers.

### Evidence synthesis

Results for the elements of the strength of association between smoking and tooth loss as well as dose-response relationship and natural experiment are summarised and evaluated using abstracted data in the table of studies (Table 2). The following levels were used to interpret the evidence for each element [12]: consistent findings among multiple high-quality studies for strong evidence, consistent findings among multiple low-quality studies and/or one high-quality study for moderate evidence, one low-quality study for limited evidence, inconsistent findings amongst multiple studies for conflicting evidence and no evidence among studies for no evidence. The present review evaluated consistency in high-quality studies.

**Table 2:** Characteristics of studies and evaluation of Newcastle-Ottawa Scale

							NOS	
							
Study design	First author, year	Participants	Age range (years)	Definition of tooth loss	Focal point	Dose-response	Coding	Score
Cross-sectional study	Randolph, 2001	3,050 Mexican American	65-99	15+	F	NA	011 11 1	5*
	Klein, 2004	2,764 American	53-96	1+	F	NA	011 11 1	5*
	Tanaka, 2005	1,002 Japanese pregnant women	29.8 on average	1+	S	NA	010 11 0	3
	Hanioka, 2007	2,200 Japanese	60-94	Total tooth loss	S	NA	101 10 1	4
	Musacchio, 2007	1,226 Italian males	65+	Total tooth loss	S	NA	101 11 1	5*
	Ojima, 2007	1,314 Japanese	20-39	1+	S	3/4 levels	111 01 1	5*
	Hanioka, 2007	3,999 Japanese	40-94	9+	S	3/4 levels	111 11 1	6*
	Mundt, 2007	2,501 German	25-59	15th percentile	F	3 levels	111 11 1	6*
	Yanagisawa, 2009	547 Japanese males	55-75	9+	S	3 levels	110 11 0	4
	Yanagisawa, 2010	1,088 Japanese males	40-75	9+	S	3 levels	110 11 0	4

							**NOS**	
							
**Study design**	**First author, year**	**Participants**	**Age range (years)**	**Duration of observation**	**Focal point**	**Dose-response**	**Coding**	**Score**

Cohort study	Slade, 1997	693 Australian	60+	2 years	F	NA	10 01 110	4
	Krall, 2006	789 American males	21-84	36 years	S	NA	00 11 111	5*
	Okamoto, 2006	740 Japanese males	30-59	4 years	S	3 levels	00 10 110	3
	Dietrich, 2007	43,112 American male health professionals	40-75	16 years	S	5 levels	01 11 111	6*
	Cunha-Cruz, 2008	12,264 American HMO members	45-61	3 years	A	NA	00 10 110	3

Studies were conducted in Japan, the United States, Australia, Germany and Italy. A dose-response relationship was examined in 7 studies, and 8 studies (6 for cross-sectional and 2 for prospective cohort studies) were classified as high quality.

S, smoking; F, factors including smoking; A, systemic antibodies; *evaluated for high-quality methodology by the modified Newcastle-Ottawa Scale (NOS). One star each was given for six and seven items for cross-sectional and cohort studies, respectively, if the methodology of a study satisfied the criterion. The items were divided according to three categories of selection, comparability and exposure for cross-sectional studies and selection, comparability and outcome for cohort studies. When a study satisfied all criteria, the star column appears as 111 11 1 for cross-sectional and 11 11 111 for cohort studies. Studies with total scores of five or more, three or four, and two or less were evaluated as high-, moderate- and low-quality studies, respectively.

Evidence synthesis was performed by considering the superiority of cohort design in reliability of evidence according to the standardised descriptions of strength of evidence for evaluating association [13], in addition to the biological plausibility of this association. The description of evidence level was categorised into four criteria as convincing, probable, possible and insufficient evidence (see Additional file 2). Due to the difficulty in conducting intervention studies, it was replaced with natural experiments [1].

## Results

### Number of studies

**Figure 1:**
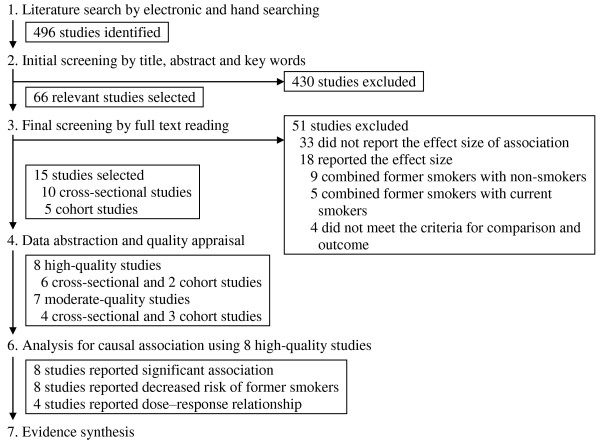
**Number of studies according to the processes of searching, selection and evaluation of literature**. The searches yielded 496 citations. The initial screening identified 66 studies, and 51 studies were excluded. Finally, 8 high-quality studies were evaluated for the causal association.

The electronic and hand searches yielded 496 citations (Figure 1). The initial screening by title, abstract and key words identified 66 relevant studies for a full-text review. Among the reviewed studies, 33 reported the effect size of association. The remaining 33 were excluded. Among the studies that reported effect size, 18 were excluded (see Additional file 3). Former smokers were evaluated with non-smokers in nine studies and with current smokers in five. Four studies did not meet the criteria for comparison and outcome. In total, we excluded 51 studies and included 15 studies.

### Characteristics and quality of studies

Ten cross-sectional and five prospective cohort studies were selected for evaluating methodological quality (Table 2). Seven studies were conducted in Japan [14-20] and five studies were reported from the United States [21-25]. Other studies were conducted in Australia [26], Germany [27] and Italy [28]. Various age groups were studied. In cross-sectional studies, the definition of tooth loss was one tooth or more or nine teeth or more in each of three studies. Total tooth loss was used in two studies, and 15 teeth or more or the 15^th ^percentile was used in another two studies. In cohort studies, the observational period was 2-36 years. Ten studies focused on smoking as the primary cause of tooth loss. Seven studies examined a dose-response relationship. According to the modified NOS, eight studies (six for cross-sectional and two for prospective cohort studies) were classified as high quality, and the remaining seven studies were categorised as moderate quality. The source of funding was public and institutional grants for seven studies (data not shown). One study was supported by a regional private foundation sector [28].

### Strength of association

The evidence of association was evaluated for each element with respect to consistency by using the abstracted data of eight high-quality studies, including two cohort studies (Table 3). These studies examined 58,755 subjects in four countries: Germany, Italy, Japan and the United States. A cross-sectional study in Germany [27] and a cohort study in the United States [24] reported effect sizes for several categories regarding exposure. The effect size for the specific category that included the median value of exposure was used. The association between current smoking and tooth loss was significant in all studies. The effect size in cross-sectional studies varied from 1.69 to 4.04. Because the prevalence of tooth loss exceeded 15% in current smokers, the strength of association was evaluated according to differences in prevalence between smoking groups. The strength of association was moderate in five studies and weak in two studies. One study indicated that the strength of association was moderate for males and weak for females. The results from high-quality studies indicated that the evidence of weak to moderate association between smoking and tooth loss was strong.

**Table 3:** Effect size of association, differences in prevalence and description of strength of association

			Association		Prevalence of tooth loss (%)			
					
Type of exposure	First author, year	Sex	Effect size (95% CI)	Type	Current smoker	Non-smoker	Difference	Strength of association
Current smokers	Randolph, 2001	M F	1.69 (1.31, 2.20)	OR	50 57	41 46	9 11	Moderate Moderate
	Klein, 2004	M, F	4.04 (2.52, 6.49)	OR	92.3	79.5	12.8	Moderate
	Musacchio, 2007	M, F	4.01 (2.59, 6.20)	OR	48.1**	42.3**	5.8**	Weak
	Ojima, 2007	M F	2.21 (1.40, 3.50) 1.70 (1.13, 2.55)	OR	39.3 43.2	21.8 29.4	17.5 13.8	Moderate Moderate
	Hanioka, 2007	M F	2.24 (1.28, 3.94) 2.74 (1.46, 5.16)	OR	36.9 38.9	28.5 38.6	8.4 0.3	Moderate Weak
	Mundt, 2007	M, F	2.3 (1.6, 3.4)*	OR	21.1**	8.4**	12.7**	Moderate
	
	Krall, 2006	M	2.1 (1.5, 3.1)	HR				Weak
	Dietrich, 2007	M	2.3 (2.1, 2.5)*	HR				Moderate

Former smokers	Randolph, 2001	M F	1.26 (1.04, 1.54)	OR	45 55	41 46	4 9	Weak*** Moderate
	Klein, 2004	M, F	1.57 (1.25, 1.98)	OR	85.8	79.5	6.3	Weak***
	Musacchio, 2007	M, F	3.42 (2.42, 4.82)	OR	45.5**	42.3**	3.2**	Weak
	Ojima, 2007	M F	1.25 (0.55, 2.86) 0.52 (0.23, 1.18)	OR	26.3 20.0	21.8 29.4	4.5 -9.4	NS***
	Hanioka, 2007	M F	1.55 (0.88, 2.74) 1.17 (0.44, 3.09)	OR	38.6 34.3	28.5 38.6	10.1 -4.3	NS***
	Mundt, 2007	M, F	1.7 (1.0, 3.1)*	OR	12.1**	8.4**	3.7**	NS***
	
	Krall, 2006	M	1.3 (0.9, 1.7)	HR				NS***
	Dietrich, 2007	M	1.2 (1.2, 1.3)*	HR				Weak***

The evidence of weak to moderate association between smoking and tooth loss was consistent in high-quality studies, and the effect size was consistently smaller for former smokers than for current smokers.

CI, confidence interval; OR, odds ratio; HR, hazard ratio; *extracted from the category that included the median value of the group as a representative because the effect size for all current or all former smokers was not reported; **calculated by reviewer based on date in the table in the original literature; ***lower rank than current smokers

### Natural experiment

The strength of association was then evaluated in former smokers in natural experiments. The association between having smoked and tooth loss was not significant in four studies. Although another four studies reported a significant association, the association was weak in three studies, and one study reported a weak association for males and a moderate association for females. The effect size was consistently smaller for former smokers than for current smokers. The strength of association was lower in former smokers than in current smokers in six studies. These results suggested that the evidence from natural experiments for evaluating the association between smoking cessation and tooth loss was strong with respect to consistency. Two cohort studies with observational periods of 16 and 36 years on populations in the United States reported decreases in hazard ratios on the basis of years of abstinence ([23,24], data not shown). One study conducted sensitivity analyses of the dentist population reporting similar trends as other health professionals with respect to the strength of association, dose-response and natural experiment [24].

### Dose-response relationship

The dose-response relationship was reported in four high-quality studies, including one cohort study (Table 4). These studies examined 50,926 subjects in three countries [16,18,24,27]. One study examined the relationship in former smokers [27]. The trend of the relationship between the level of exposure and effect size, i.e. odds ratio or hazard ratio, was obvious in all studies. The dose-response relationship was significant in two studies. Therefore, the evidence for a dose-response relationship between smoking and tooth loss was also strong with respect to consistency.

**Table 4:** Relationship between exposure of smoking and effect size

Author, year	Unit of exposure	Smoking status	Sex	Exposure Effect size						P for trend
Ojima, 2007	Pack-years	Current	M	1-9 1.34*	10-19 2.75	20- 5.17				<0.0001
		Current	F	1-9 1.31*	10- 5.34					0.0004
Hanioka, 2007	Pack-years	Current	M	1-19 1.99	20-59 2.20	60- 2.94				<0.0001
		Current	F	1-19 1.74	20-59 2.30	60- 14.5				<0.0001
Mundt, 2007	Daily consumption	Current	M, F	1-9 2.1	10-19 2.3	20- 3.5				NA
		Former	M, F	1-9 1.3*	10-19 1.7	20- 2.4				NA

Dietrich, 2007	Daily consumption	Current	M	1-4 1.4	5-14 1.9	15-24 2.3	25-34 2.7	35-44 2.9	45- 3.0	NA

Trend of the relationship between the level of exposure and effect size was obvious in all studies.

*Not significant

### Evaluation of evidence

The results from the assessment of each element suggested that the evidence was strong in terms of consistency (Table 5). This interpretation was based on consistent results with little or no evidence to the contrary in six cross-sectional and two prospective cohort studies. Prospective observational design is generally considered a more reliable study design than a cross-sectional design. The inclusion of cohort studies indicates more convincing evidence of causal association. However, natural experiments were carried out relative to non-smokers, and both natural experiments and dose-response relationships were reported for a limited number of countries. These shortcomings necessitated the downgrading of the description from 'convincing' to 'probable' for the overall strength of evidence of causal association between smoking and tooth loss.

**Table 5:** Summary of results to evaluate the causal association between smoking and tooth loss

Element	Description for consistency and study type	Evaluation of consistency	Evaluation of study type	Perceived shortcoming
Strength of association	All studies reported significant associations based on effect size: moderate association in 5 CSSs, 1 PCS and 1 CSS for males; weak association in 1 PCS and 1 CSS for females.	Evidence for weak or moderate association based on effect size is strong.	Evidence for strength of association is convincing.	Not applicable
Natural experiment	All studies reported smaller effect size in former smokers than in current smokers. The association between former smoking and tooth loss relative to non-smokers was not significant in 3 CSSs and 1 PCS, and was significant in 3 CSSs and 1 PCS. The description of association in former vs. current smokers decreased in 4 CSSs, 2 PCSs and 1 CSS for males, and remained at the same level in 1 CSS and 1 CSS for females. The hazard ratio decreased based on years of abstinence in 2 PCSs.	Evidence for natural experiment is strong. However, this interpretation does not mean that the risk in former smokers is lesser than that in current smokers.	Evidence for natural experiment is probable.	Control group did not comprise current smokers, and only a relative relationship was evaluated.
Dose-response relationship	Trend of the relationship between level of exposure and effect size, i.e. odds ratio or hazard ratio, was obvious in 3 CSSs and 1 PCS. This trend was highly significant in 2 CSSs.	Evidence for the dose-response relationship is strong. This interpretation is limited to populations assessed in 3 countries.	Evidence for dose-response relationship is probable.	Findings pertain to limited populations and 1 PCS.

Evaluation in each element was based on the consistency of findings and study types.

CSS: cross-sectional study, PCS: prospective cohort study

## Discussion

Published literature was reviewed and screened systematically, and eight studies met the criteria for the high-quality category. The evidence for each element inferring a causal association between smoking and tooth loss in high-quality studies was summarised and evaluated on the basis of standardised methodologies with respect to consistency and study design. The evidence supporting this causal association was consistent. The association cannot be explained by confounding factors. Several concerns regarding the reporting of the synthesis of evidence will be addressed before the final description of the overall evidence.

First, the validated association in the epidemiologic literature should be biologically plausible. The most plausible biological connection between substances in tobacco smoke and tooth loss is the destruction of tooth-supporting tissue. Previous studies have shown several pathways on the basis of exisisting knowledge of the effects on the entire body of smoking [1]. These include dysfunction of gingival fibroblasts, a decrease in microcirculatory function and immune system deficiency. Periodontal destruction in smokers may be modulated by an impaired ability to repair damaged tissue rather than by direct tissue damage. Recent progress in molecular and genetic approaches have made a deeper exploration of the mechanisms possible [29]. In a previous study, smokers exhibited overproduction of inflammatory molecules and suppression of anti-inflammatory molecules, thereby leading to inflammatory destruction of connective tissue and alveolar bone, though evidence of interaction with genetic factors is inconsistent.

A series of recent studies [30-32] revealed a bacteriological mechanism by utilising a novel methodology for bacterial identification. The microbial profile of disease-associated and health-compatible organisms in smoking-associated periodontitis patients was significantly different from that in non-smokers. Following nonsurgical periodontal therapy and smoking cessation counselling, those who continued smoking had a microbial profile similar to the baseline, while the subgingival microbiome in those who stopped smoking exhibited a healthy profile. These findings explain the connection between smoking and periodontal tissue breakdown by pathogenic periodontal micoorganisms.

The effects of several chemicals in tobacco smoke on the immune system and tissue repair in relation to periodontal tissue breakdown have been reported [1,33]. Nicotine, benzo(a)pyrene and benzo(a)anthracene are immunosuppressive, whereas tobacco glycoprotein and metals are immunostimulatory. Exposure to hydrocarbons could modulate immune response. Nicotine and some other tobacco compounds such as acrolein and acetaldehyde inhibit the function of gingival fibroblasts, including proliferation, collagen production, adhesion to root surfaces and induce cytotoxicity. Together, the substantive evidence strongly supports the biological plausibility of these effects.

The NOS evaluated the methodological considerations for various biases, but other sources may be considered. Early death in current smokers that have lost more teeth than non-smokers could dilute the effect of smoking in the elderly, particularly in studies that employ total tooth loss as the outcome measure. Only one study reported the dropout rate in the entire cohort population [24], and no other study accounted for the possibility of participation bias between comparison groups in identical cross-sectional samples. Smokers with fewer teeth may not have participated in the study compared with non-smokers with more teeth, leading to the underestimation of the effect of smoking.

Publication bias based on the finding of a significant association is inevitable in a literature review. Although associations between smoking and tooth loss were focused on in five high-quality studies, three other studies that reported significant associations examined various other factors, which may weaken the existence of a publication bias.

In Japanese studies, 20 existing teeth or more was used as the definition of tooth loss [16,19,20], because '20 existing teeth till 80 years' was set as an objective of the national oral health promotion programme. A definition based on malfunction may more accurately reflect the risk of smoking than one lost tooth or total tooth loss. In a cross-sectional study, the 15th percentile for each age group was employed as the definition of tooth loss [27]. This method may help to decrease heterogeneity in the definition of tooth loss due to differences in study populations in terms of the variety of tooth-loss profiles. Study funding can also be an important source of heterogeneity, but all studies were supported by public or quasi-public grants.

Distribution of the level of exposure within groups of current or former smokers may vary according to the study population; for example, health professionals may have stopped smoking many years ago [24]. Observed differences in effect size could be explained in part by the difference in distribution of exposure level. The results reported for populations in four countries where smoking has been prevalent strongly support a causal association at the population level. Because effects of smoking generally appear in later life, reports from countries where smoking rates are increasing are expected. Unfortunately, reports from such countries were excluded due to concerns regarding methodological quality (data not shown). Further studies with high-quality methodology that use data from populations in countries where smoking rates are increasing are necessary.

In the present review of cross-sectional studies, qualitative evaluation was based on differences in the prevalence of tooth loss. Because the difference in prevalence was not adjusted for any possible confounder, the results of qualitative evaluation should be interpreted carefully. For example, the lack of adjustment for age underestimates differences in the prevalence of tooth loss because of the increasing prevalence of tooth loss in a decreasing number of current smokers with age. The hazard ratio, which considers the observational period of each case in a cohort study, is the most accurate indicator of the effect size [23,24]. We did not use unpublished material and articles written in languages other than English or contact authors of original studies. The effect sizes in two studies were represented by the data of specific categories. These issues may be limitations on the interpretation of the abstracted data.

Randomised controlled studies are scarce because of difficulties associated with smoking cessation and the need for long-term observations. Natural experiments on the decreased risk in former smokers over time after stopping smoking could provide reliable secondary evidence of causal associations in observational studies. Cohort studies have revealed that longer periods of smoking cessation are associated with a lower risk of tooth loss in a dose-response manner [23,24]. The findings of a decreasing risk of tooth loss with increasing time since stopping smoking may strengthen the interpretation of causal association. Further studies with data obtained from longitudinal cohorts should be conducted among populations from countries other than the United States.

## Conclusions

A causal association between smoking and tooth loss was evaluated using high-quality studies that employed rigorous approaches. The evidence for each element supporting the causal association between smoking and tooth loss is consistently strong, but there are some shortcomings with respect to study type and population. Based on the consistent evidence of each element for evaluating this causal association with existing biological plausibility, the evidence supporting a causal association between smoking and tooth loss appears to be strong. Further studies with data obtained from the prospective cohort design and in populations from countries where smoking rates are increasing are necessary to confirm this association.

## Competing interests

The authors declare that they have no competing interests.

## Authors' contributions

TH searched and evaluated literature and organised manuscript. MO, KT and FS searched and evaluated the literature. KM organised the reviewing process. HT organised discussions. All authors read and approved the final manuscript.

## Pre-publication history

The pre-publication history for this paper can be accessed here:

http://www.biomedcentral.com/1471-2458/11/221/prepub

## Supplementary Material

Additional file 1 **Journals used for hand-search**. Click here for file

Additional file 2 **Criteria used to describe the strength of evidence of relationship**. Click here for file

Additional file 3 **Literature excluded for quality assessment by reasons for exclusion**. Click here for file
