# Supplementary material for: Causal assessment of smoking and tooth loss: A systematic review of observational studies
Source: BMC Public Health. 2011 Apr 8;11:221. doi: 10.1186/1471-2458-11-221 (PMC3087682; doi:10.1186/1471-2458-11-221)
Supplement: Additional file 1 — Journals used for hand-search. [file 1471-2458-11-221-S1.DOC]

### Additional file 1 – Journals used for hand-search

| Community Dentistry and Oral Epidemiology  Gerodontology  Journal of Clinical Periodontology  Journal of Dental Research  Journal of Periodontology  Journal of Periodontal Research  Journal of Public Health Dentistry  Oral Diseases |
| --- |
