# Supplementary material for: Causal assessment of smoking and tooth loss: A systematic review of observational studies
Source: BMC Public Health. 2011 Apr 8;11:221. doi: 10.1186/1471-2458-11-221 (PMC3087682; doi:10.1186/1471-2458-11-221)
Supplement: Additional file 2 — Criteria used to describe the strength of evidence of relationship. [file 1471-2458-11-221-S2.DOC]

### Additional file 2 – Criteria used to describe the strength of evidence of relationship

| Convincing evidence | Evidence is based on epidemiological studies showing consistent associations between exposure and disease, with little or no evidence to the contrary. The available evidence is based on a substantial number of studies including prospective observational studies and where relevant, randomized controlled trials of sufficient size, duration and quality showing consistent effects. The association should be biologically plausible. |
| --- | --- |
| Probable evidence | Evidence is based on epidemiological studies showing fairly consistent associations between exposure and disease, but where there are perceived shortcomings in the available evidence or some evidence to the contrary, precluding a more definite judgment. Shortcomings in the evidence may be any of the following: insufficient duration of trials (or studies); insufficient trials (or studies) available; inadequate sample sizes; and incomplete follow-up. Laboratory evidence is usually supportive. Again, the association should be biologically plausible. |
| Possible evidence | Evidence is based mainly on the findings from case-control and cross-sectional studies. Insufficient randomized controlled trials, observational studies or nonrandomized controlled trials are available. Evidence based on non-epidemiological studies, such as clinical and laboratory investigations, is supportive. More trials are required to support the tentative associations, which should also be biologically plausible. |
| Insufficient evidence | Evidence is based on the findings of a few studies which are suggestive, but are insufficient to establish an association between exposure and disease. Limited or no evidence is available from randomized controlled trials. More well-designed research is required to support the tentative associations. |

Nishida C, Uauy R, Kumanyika S, Shetty P: **The Joint WHO/FAO Expert Consultation on diet, nutrition and the prevention of chronic diseases: process, product and policy implications.** *Public Health Nutr* 2004, **7**: 245-250.
