# Supplementary material for: Causal assessment of smoking and tooth loss: A systematic review of observational studies
Source: BMC Public Health. 2011 Apr 8;11:221. doi: 10.1186/1471-2458-11-221 (PMC3087682; doi:10.1186/1471-2458-11-221)
Supplement: Additional file 3 — Literature excluded for quality assessment by reasons for exclusion. [file 1471-2458-11-221-S3.DOC]

### Additional file 3 – Literature excluded for quality assessment by reasons for exclusion

| **Former smokers were combined with non-smokers.**  Atieh MA: **Tooth loss among Saudi adolescents: social and behavioural risk factors.** *Int Dent J* 2008, **58**: 103-108.  Chambrone LA and Chambrone L: **Tooth loss in well-maintained patients with chronic periodontitis during long-term supportive therapy in Brazil.** *J Clin Periodontol* 2006, **33**: 759-764.  Chatrchaiwiwatana S: **Factors affecting tooth loss among rural Khon Kaen adults: Analysis of two data sets.** *Public Health* 2007, **121**: 106-112.  Fardal Ø, Johannessen AC, Linden GJ: **Tooth loss during maintenance following periodontal treatment in a periodontal practice in Norway.** *J Clin Periodontol* 2004, **31**: 550-555.  Jette AM, Feldman HA, Tennstedt SL: **Tobacco use: a modifiable risk factor for dental disease among the elderly.** *Am J Public Health* 1993, **83**: 1271-1276.  Krall EA, Dawson-Hughes B, Garvey AJ, Garcia RI: **Smoking, smoking cessation, and tooth loss.** *J Dent Res* 1997, **76**: 1653-1659.  Krall EA, Garvey AJ, Garcia RI: **Alveolar bone loss and tooth loss in male cigar and pipe smokers.** *J Am Dent Assoc* 1999, **130**: 57-64.  Lawton B, Rose S, Kieser J, Broadbent J, Sussex P, Thomson M, Dowell A: **Disparities in edentulism and tooth loss between Maori and non-Maori New Zealand women.** *Aust N Z J Public Health* 2008, **32**: 254-260.  Worthington H, Clarkson J, Davies R: **Extraction of teeth over 5 years in regularly attending adults.** *Community Dent Oral Epidemiol* 1999, **27**: 187-194. |
| --- |
| **Former smokers were combined with current smokers.**  Suominen-Taipale Al, Alanen P, Helenius H, Nordblad A, Uutela A: **Edentulism among Finnish adults of working age, 1978-1997.** *Community Dent Oral Epidemiol* 1999, **27**: 353-365.  Xie Q and Ainamo A: **Association of edentulousness with systemic factors in elderly people living at home.** *Community Dent Oral Epidemiol* 1999, **27**: 202-209.  Ylöstalo P, Sakki T, Laitinen J, Järvelin MR, Knuuttila M: **The relation of tobacco smoking to tooth loss among young adults.** *Eur J Oral Sci* 2004, **112**: 121-126.  Susin C, Haas AN, Opermann RV, Albandar JM: **Tooth loss in a young population from south Brazil.** *J Public Health Dent* 2006, **66**: 110-115.  Moedano DE, Irigoyen ME, Borges-Yáňez, Flores-Sánchez I, Rotter RC: **Osteoporosis, the risk of vertebral fracture, and periodontal disease in an elderly group in Mexico City.** *Gerodontology* 2009, Article first published online: 26 OCT 2009. |
| **Tooth loss was compared with reasons for extraction by smoking status.**  Al-Shammari KF, Al-Khabbaz AK, Al-Ansari JM, Neiva R, Wang HL: **Risk indicators for tooth loss due to periodontal disease.** *J Periodontol* 2005, **76**: 1910-1918. |
| **Tooth loss was combined with periodontal attachment loss.**  Kocher T, Schwahn C, Gesch D, Bernhardt O, John U, Meisel P, Baelum V: **Risk determinants of periodontal disease – an analysis of the study of health in Pomerania (SHIP 0).** *J Clin Periodontol* 2005, **32**: 59-67. |
| **Tooth loss was defined by three levels.**  Susin C, Oppermann RV, Haugejorden O, Albandar JM: **Tooth loss and associated risk indicators in an adult urban population from south Brazil.** *Acta Odontol Scand* 2005, **63**: 85-93. |
| **Prosthetic treatment was used for outcome measure.**  Zitzmann NU, Staehelin K, Walls AWG, Menghini G, Weiger R, Zemp Stutz E: **Changes in oral health over a 10-yr period in Switzerland.** *Eur J Oral Sci* 2008, **116**: 52–59. |
